# α_S1_-casein, which is essential for efficient ER-to-Golgi casein transport, is also present in a tightly membrane-associated form

**DOI:** 10.1186/1471-2121-11-65

**Published:** 2010-08-12

**Authors:** Annabelle Le Parc, Joëlle Leonil, Eric Chanat

**Affiliations:** 1 INRA, UR1196 Génomique et Physiologie de la Lactation, Domaine de Vilvert, F-78352 Jouy-en-Josas cedex, France; 2 INRA, UMR1253 Science et Technologie du Lait et de l'Œuf, F-35000 Rennes, France

## Abstract

**Background:**

Caseins, the main milk proteins, aggregate in the secretory pathway of mammary epithelial cells into large supramolecular structures, casein micelles. The role of individual caseins in this process and the mesostructure of the casein micelle are poorly known.

**Results:**

In this study, we investigate primary steps of casein micelle formation in rough endoplasmic reticulum-derived vesicles prepared from rat or goat mammary tissues. The majority of both α_S1_- and β-casein which are cysteine-containing casein was dimeric in the endoplasmic reticulum. Saponin permeabilisation of microsomal membranes in physico-chemical conditions believed to conserve casein interactions demonstrated that rat immature β-casein is weakly aggregated in the endoplasmic reticulum. In striking contrast, a large proportion of immature α_S1_-casein was recovered in permeabilised microsomes when incubated in conservative conditions. Furthermore, a substantial amount of α_S1_-casein remained associated with microsomal or post-ER membranes after saponin permeabilisation in non-conservative conditions or carbonate extraction at pH11, all in the presence of DTT. Finally, we show that protein dimerisation via disulfide bond is involved in the interaction of α_S1_-casein with membranes.

**Conclusions:**

These experiments reveal for the first time the existence of a membrane-associated form of α_S1_-casein in the endoplasmic reticulum and in more distal compartments of the secretory pathway of mammary epithelial cells. Our data suggest that α_S1_-casein, which is required for efficient export of the other caseins from the endoplasmic reticulum, plays a key role in early steps of casein micelle biogenesis and casein transport in the secretory pathway.

## Background

During lactation, mammary epithelial cells (MECs) secrete huge quantities of milk-specific proteins and other components such as lipids and lactose. The main milk proteins (except in primates) are the caseins, a family of acidic phosphoproteins (α_S1_-, α_S2_-, β- and κ-casein; for review see [1]) the proportions of which vary widely across species and occasionally among animals of the same species. Caseins interact with calcium and calcium phosphate, and self-aggregate to organize into a supramolecular structure known as the casein micelle. The central physiological function of the casein micelle is to supply proteins, phosphate and calcium to neonates.

The mesostructure of the micelle determines the techno-functional characteristics of the milk protein fraction and has an impact on milk processing. Despite the importance of the nutritional and functional values of casein micelles, which justifies many years of intense research (for review see [2-4]), the detailed intrinsic organisation and the mechanisms involved in the formation of this structure have not been fully established. Although casein micelles vary in size, compactness, protein and mineral compositions, their structure as a whole is believed to be similar across species, implying that very general features are involved in their biogenesis. Several conflicting models of the internal structure of casein micelles have emerged, largely from morphological observations, biochemical and physical studies in vitro. In the submicelle model, which was the most accepted for many years, caseins are clustered into small spherical subunits which are further linked together by calcium phosphate (for review see [5]). An alternative model, first proposed by Holt (for review see [2]) and extended by Horne [6], is the tangled web model. In this model, caseins self-associate, mainly through hydrophobic and electrostatic interactions, to form a homogeneous network of casein polymers stabilized through interaction with nanoclusters of calcium phosphate. It follows that the small substructures observed within casein micelles at the electron microscopy level or detected by Small Angle X-ray Scattering as a characteristic point of inflection in SAXS profiles might well be calcium phosphate nanoclusters rather than submicelles [7]. In both models, κ-casein, which is highly glycosylated, preferentially localizes at the periphery of the micelle and forms a layer at the protein-water interface, stabilizing the structure and preventing it from aggregating.

The four major caseins are heterogeneous, genetic polymorphisms and variations in post-translational modifications reinforcing diversity in a given species. This is the case in goat, for example, due to an extensive polymorphism at the *CSN1S1 *casein locus [8]. It is also clear that very little of the primary sequence of each of the caseins is fully conserved, making the caseins one of the most evolutionarily divergent families of mammalian proteins. Despite this high heterogeneity of components, casein micelles are found in all mammalian milks as far as we know and seem quite similar at the ultra structural level. They also form in the absence of α_S1_- or β-casein [9,10]. Interactions between the various caseins and minerals during micelle biogenesis might therefore involve rather general physico-chemical and biochemical characteristics of these components. However, these characteristics are specific enough to avoid incorporation of whey proteins in the micelle. In agreement with these considerations, caseins were shown to lack appreciable amount of regular secondary structure. β-and κ-casein might possess pre-molten or molten globule conformations whereas α_S1_- and α_S2_-casein are predicted intrinsically unstructured proteins [11] (or natively unfolded proteins, [12]). The characteristic structural feature of natively unfolded proteins is a combination of low mean hydrophobicity and relatively high proportion of charged residues at physiological pH [13]. Proteins with such open structure possess a peculiar aggregative behaviour and are prone to interact with their specific ligand in vivo. The caseins, however, do not fulfil these two criteria since they present relatively high hydrophobicities.

First interactions between caseins obviously take place in the endoplasmic reticulum (ER). In all species for which the primary sequence has been determined, κ-casein contains at least one cysteine in its N-terminal domain [14]. Hence, it can form dimers via disulphide bond linkage, a post-translational modification that occurs in the ER. It has been shown that native bovine κ-casein forms dimers and multimers [15], whereas rodent milk κ-casein is seemingly only present as dimers [14]. Depending on the species, cysteine residues are also found in other casein sequences, notably in α_S2_-casein. An extreme case is the rat, in which all caseins contain one cysteine (except δ-casein which possess two) and are dimeric in milk [14]. Disulphide bond formation, which is one of the first steps in protein maturation, could therefore play a key role in casein aggregation and in the elaboration and stability of the casein micelle structure.

While studying the impact of the polymorphism at the *CSN1S1 *locus on goat milk secretion, we showed that, in the absence of α_S1_-casein, other caseins accumulate in the ER [9]. Kinetic analysis revealed that the efficiency of casein transport from the ER to the Golgi apparatus was strongly affected in this context. Our data suggested that interaction of caseins in a yet to be identified structure including α_S1_-casein is required for efficient transport of these proteins to the Golgi apparatus. During their traffic through the Golgi cisternae, all caseins are phosphorylated to various extents and κ-casein is O-glycosylated. Phosphorylation allows calcium phosphate binding and further interactions between caseins, a key step in the formation of casein micelles. Notably, the phosphorylation of β-casein seems delayed compared to that of α_S1_-casein [16-18]. This suggests that strong interaction of this protein with casein polymers might be postponed until it is trafficked to trans Golgi cisternae. Consistent with these results, premicellar aggregates have been observed by electron microscopy in the lumen of the Golgi cisternae. There is a drastic rearrangement of the micellar structure during formation of secretory vesicles at the trans side of the Golgi apparatus and their transport to the apical plasma membrane for exocytosis [19]. In newly formed transport vesicles, caseins are under the form of long loose linear aggregates [19,20]. These progressively self-associate, become bigger and denser, and structures with the characteristic honeycomb texture of casein micelles are found in distal secretory vesicles. These structures are similar, if not identical, to those observed in the lumen of the acini.

This biochemical and morphological information suggests that casein aggregation is initiated in the ER and gradually proceeds during their transport to the apical surface. We believe we must exploit the spatio-temporal dimension of casein micelle biogenesis and study their formation within the secretory pathway of MECs to obtain new insights into casein micelle structure. With this aim, we investigated primary steps of casein interaction in the rough ER.

## Results

To study the state of aggregation of the caseins in the secretory pathway of MECs we chose the rat mammary gland. Rat milk contains a high concentration of milk proteins, including 70 mg/ml of caseins [21]. Rat casein micelles are easily pelletable and there are no detectable non-micellar caseins in milk, as is observed in certain species. We have previously identified casein dimers in rat milk [14], as all rat caseins contain at least one cysteine. Finally, we have characterised the various molecular forms of rat α_S1_- and β-casein. These include the immature ER forms, which are not yet phosphorylated, and the mature phosphorylated forms, which occur as from the Golgi apparatus [17,22].

### Properties of the rough microsomal fraction

**Figure 1:**
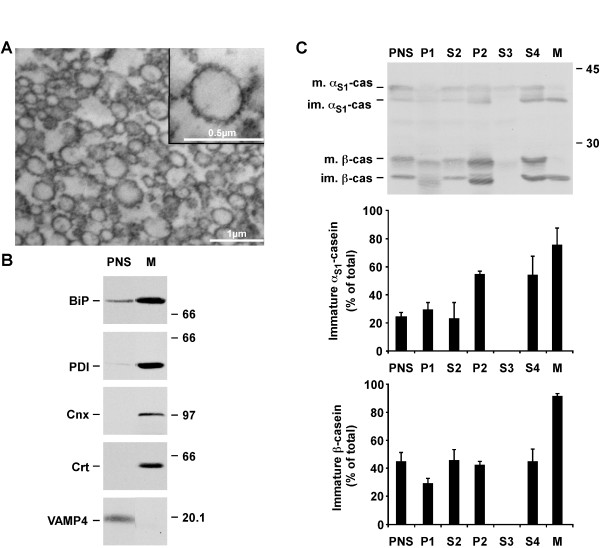
**The rough ER microsomal fraction from a lactating rat is almost pure**. The rough ER microsomal fraction (M) was prepared from a post-nuclear supernatant (PNS) of mammary tissue from lactating animals by differential centrifugation followed by sucrose density gradient. A. An aliquot of the ER microsomal fraction was fixed and processed for electron microscopy. Insert shows a microsome associated with numerous ribosomes. B. Aliquots of PNS and of the ER microsomal fraction were analyzed by SDS-PAGE followed by immunoblotting for the indicated protein markers. Representative ECL signals from two independent experiments are shown. C. Aliquots of the different supernatants (PNS, S) and pellets (P, M) collected during the purification (see Methods for detailed identification of the fractions) were analyzed by SDS-PAGE followed by immunoblotting using an antibody against mouse milk proteins. The amount of immature forms of α_S1_- and β-caseins was quantified by densitometry and expressed as percent of the total quantity of the individual casein (mature + immature). The mean ± s.d. of three independent experiments is shown. Relative molecular masses (kDa) are indicated on the right of the immunoblots. α_S1_-cas: α_S1_-casein; β-cas: β-casein; BiP: immunoglobulin heavy chain binding protein; Cnx: calnexin; Crt: calreticulin; im.: immature; m.: mature; VAMP4: vesicle-associated membrane protein 4.

The rough ER microsomal fraction from rat mammary gland cells was obtained by differential centrifugation followed by sucrose density gradient, essentially as described by Paiement et al. [23]. Some modifications of the original protocol were necessary to adapt the method to mammary gland tissue and to eliminate some contaminants such as mitochondria and, more importantly, casein micelles derived from the milk contained in the acini and ducts of the tissue. Morphological observation indicated that the rough ER microsomal fraction was almost pure (Figure 1A). The insert shows that, as expected, most if not all microsomes were associated with numerous electron-dense ribosomal particles. Quantitative analysis revealed that the size of the microsomes was heterogeneous, with a mean diameter of 0.15 ± 0.03 μm and diameters ranging from 0.07 to 0.36 μm. These results were in agreement with those observed by Lavoie et al. [24] using rat liver. Purification of the rough ER-derived membrane-bounded vesicles in the heavy microsomal fraction was further studied by immunoblotting. Figure 1B shows that several ER resident proteins, namely immunoglobulin heavy chain binding protein (GRP78/BiP), protein disulphide isomerase (PDI), calnexin (Cnx) and calreticulin (Crt), were enriched in the heavy microsomal fraction, as compared to the post-nuclear supernatant (PNS). In contrast, vesicle-associated membrane protein 4 (VAMP4) and GM130 (data not shown), two markers of the Golgi apparatus, were not detected in these membranes.

Analysis of the distribution of the mature and immature forms of caseins in the various fractions collected during subcellular fractionation confirmed the purification of the rough ER-derived vesicles (Figure 1C). The relative proportion of the immature forms of α_S1_- and β-casein was greatly increased in the microsomal fraction. Densitometric analysis indicated that immature forms were 2-3 times more abundant in the microsomal pellet than in the PNS. Altogether, these data demonstrated the high enrichment of rough ER-derived vesicles in the heavy microsomal fraction.

### Caseins primarily interact via disulphide bonds in the rough ER

**Figure 2:**
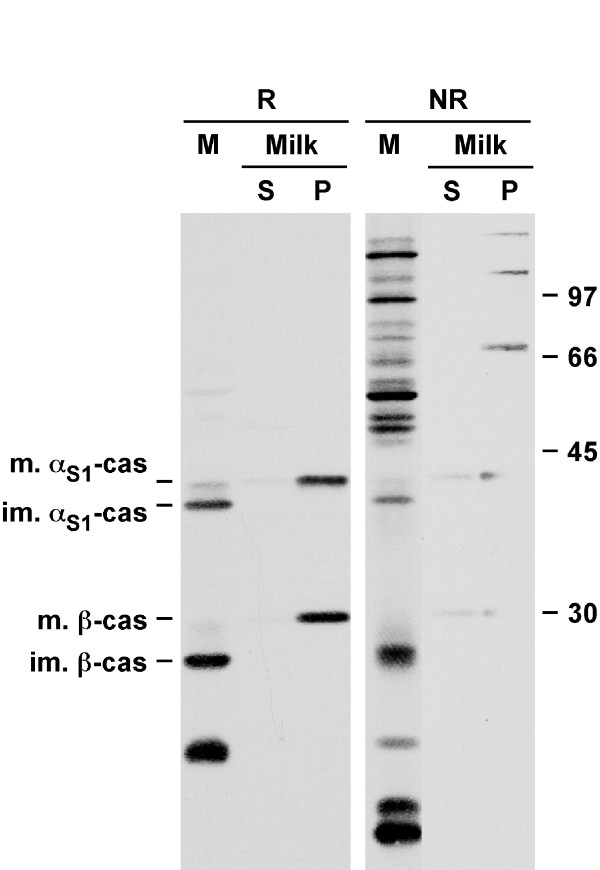
**Caseins in rat rough ER microsomes are mostly dimeric**. Aliquots of the aqueous phase of rat milk were diluted and centrifuged at room temperature. Milk supernatant (S), pellet (P), and an aliquot of the rough ER microsomal fraction (M), were analyzed by SDS-PAGE in reducing (R) or non-reducing (NR) conditions followed by immunoblotting using an antibody against mouse milk proteins. Relative molecular masses (kDa) are indicated on the right of the immunoblots. α_S1_-cas: α_S1_-casein; β-cas: β-casein; im.: immature; m.: mature

Since we had previously established that cysteine-containing caseins, notably rat caseins, form disulphide bonds and are dimeric in milk, it was important to determine whether such dimers exist in the ER of rat MECs. Aliquots of rough ER microsomes were analyzed by immunoblotting under reducing or non-reducing conditions and the milk protein patterns were compared to those obtained with caseins from rat milk. As previously reported [14], in the absence of β-mercaptoethanol, mature monomeric forms of α_S1_- and β-casein in a casein pellet prepared from skim milk disappeared, and these proteins were detected at much higher Mr than in reducing conditions (Figure 2). Accordingly, when microsomes were analyzed in non-reducing conditions, the amount of immature monomeric forms of α_S1_- and β-casein was decreased (Figure 2, compare M in reducing and non-reducing conditions) and several bands appeared at the top of the gel. These data indicated that a substantial part of the caseins were in dimeric forms in the ER of rat MECs.

### α_S1_-casein remains in permeabilised rough ER microsomes after incubation in conservative conditions

To further determine the types of interactions that exist between caseins in the ER, we chose to permeabilise microsomal membranes with saponin to gain access to the lumen, as was previously reported for Golgi and secretory granule membranes [25]. Since the cholesterol concentration in the ER is quite low [26], we had to use twice as much saponin (0.1%) to efficiently release soluble ER proteins. Membrane permeabilisation was in conditions believed either to conserve or not to conserve the aggregated state of the caseins. It is well known that micelle stability is highly dependent on several physico-chemical parameters [27] including temperature, salt and calcium concentrations, and pH. Thus, non-conservative conditions were a slightly basic pH (7.4), the presence of a calcium chelator (20 mM EDTA) and salts (150 mM NaCl), plus a small quantity of detergent (0.3% Tween 20) and of a reducing agent (5 mM DTT). In conservative conditions all of these destabilizing agents were omitted and only a low ionic strength buffer was used (10 mM Hepes buffer pH 6.8, the pH of milk).

**Figure 3:**
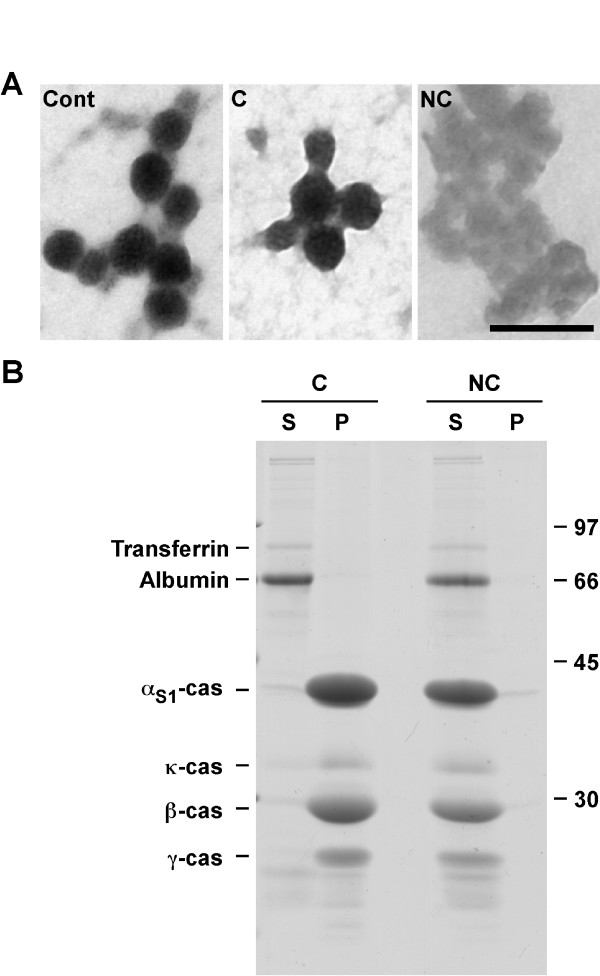
**Recovery of casein micelles after incubation in conservative conditions**. Aliquots of the aqueous phase of rat milk were diluted and incubated in conservative (C) or non-conservative (NC) buffers. A. Samples were processed for negative staining electron microscopy. An undiluted aliquot of the aqueous phase of rat milk was directly analyzed (Cont). Scale bar: 0.2 μm. B. Samples in conservative or non-conservative buffers were centrifuged. Supernatants (S) and pellets (P) were analyzed by SDS-PAGE followed by Coomassie Blue staining. Relative molecular masses (kDa) are indicated on the right. Data are representative of at least three independent experiments. α_S1_-cas: α_S1_-casein; β-cas: β-casein; κ-cas: κ-casein; γ-cas: γ-casein.

First, we tested these conditions on casein micelles from milk. Negative staining in electron microscopy showed that, after dilution and incubation in conservative buffer, the casein micelle structure was preserved (Figure 3A). In contrast, incubation in non-conservative conditions resulted in the disappearance of the typical spherical aspect of the micelle and to a high decrease in the electron density of the sample. In line with these morphological results, when casein micelles were diluted and incubated in conservative conditions, they were recovered in the pellet after centrifugation whereas all caseins were found in the supernatant when non-conservative conditions were used (Figure 3B). Analysis of rat milk caseins incubated in conservative conditions plus 100 mM NaCl using negative staining or sedimentation on a linear sucrose gradient confirmed that salt was deleterious (data not shown). We concluded that the conservative and non-conservative conditions were effective in preserving or destroying the micellar structure, respectively.

**Figure 4:**
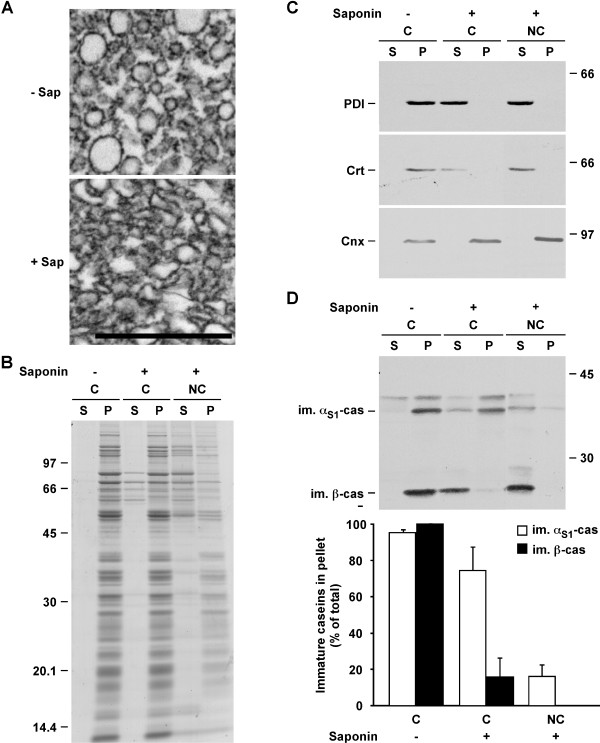
**The majority of α_S1_-casein, but not of β-casein, remains in the ER after saponin permeabilisation of microsomal membranes in conservative conditions**. A. Electron micrographs of microsomes incubated in conservative conditions in the absence (- Sap) or the presence (+ Sap) of saponin and centrifuged. The membrane pellet was fixed and processed for electron microscopy. B-D. Aliquots of the microsomes were diluted and incubated in conservative (C) buffer in the absence of saponin (-), or in conservative or non-conservative (NC) buffers in the presence of saponin (+). After centrifugation, supernatants (S) and pellets (P) were analyzed by SDS-PAGE. B. Coomassie Blue staining. C. Immunoblotting using antibodies against the indicated ER-resident proteins. D. Immunoblotting with an antibody against mouse milk proteins. Immature α_S1_- and β-casein were quantified by densitometry and the proportion of the immature form in the pellet was expressed as percent of the total quantity of the casein (supernatant + pellet). The mean ± s.d. of three independent experiments is shown. Relative molecular masses (kDa) are indicated. Scale bar: 1 μm. Cnx: calnexin; Crt: calreticulin; im. α_S1_-cas: immature α_S1_-casein; im. β-cas: immature β-casein.

We then wished to expose the caseins present within the ER lumen to the conditions used above by means of saponin permeabilisation of the heavy microsomal membranes. However, we first tested whether saponin has deleterious effects on casein aggregates, by analyzing its effect on casein micelles from milk in conservative conditions. Both negative staining and differential centrifugation of diluted milk showed that the standard concentration of saponin used in our experiments had no detectable impact on casein micelle structures (data not shown). We also verified that saponin did not solubilize the microsomal membranes. It should be noted that for analysis of casein aggregates in the ER we use pH 7.0 for conservative conditions, which is the pH believed to exist in the ER lumen [28]. Aliquots of the microsomal fraction were incubated in conservative conditions in the absence or in the presence of saponin, centrifuged and processed for electron microscopy. Morphological analysis revealed that rough ER microsomes lost their spherical shape in the presence of saponin but that the detergent did not destroy the microsomal membranes (Figure 4A). After thawing and incubation of microsomal membranes in conservative conditions and subsequent centrifugation, a very small amount of protein was detected in the supernatant (Figure 4B). When saponin (0.1%) was added to the conservative buffer, most of the proteins were still associated with the membrane pellet but several proteins were found in substantial amounts in the supernatant. Certain of these proteins, notably those migrating between ≈ 55,000 and ≈ 95,000, most likely corresponded to soluble ER-resident proteins (see below). When non-conservative conditions were used, the amounts of some of these proteins were slightly increased and additional bands were recovered in the supernatant. We tried to identify these proteins by mass spectrometry (LC MS/MS). However, definitive identification of the proteins contained in the gel slices was hampered by the high quantity of proteins present in each gel slice (data not shown). To further characterise this experimental system, we studied several well-known ER markers by immunoblotting (Figure 4C). In the absence of saponin, all of these ER-resident proteins were indeed recovered in the pellet. Permeabilisation of microsomal membranes in both conservative and non-conservative conditions induced the complete release of PDI and calreticulin, two soluble ER proteins. In striking contrast, calnexin was only found in the pellet in both conditions, in agreement with the fact that this ER marker has a transmembrane domain. These results, together with our morphological observations, demonstrated that 0.1% saponin allows for the efficient permeabilisation of microsomal membranes and for the release of soluble proteins.

The levels of solubility of immature caseins were therefore studied after saponin permeabilisation of microsomal membranes. Most of the α_S1_-casein (74.5%) was found in the pellet in conservative conditions (Figure 4D). In contrast, only 15.9% of β-casein remained in the pellet in these conditions. In non-conservative conditions, which are expected to disorganize potential casein aggregates in the ER lumen, the vast majority of the caseins were indeed released from permeabilised ER vesicles and recovered in the supernatant. It should be noted, however, that a substantial amount of α_S1_-casein remained associated with the membrane pellet (16.14%). From these data we concluded that most α_S1_-casein appeared to be aggregated in the lumen of the microsomes. Moreover, our results suggest that a part of the α_S1_-casein was associated with the ER membranes.

### α_S1_-casein is present in a membrane-associated form

**Figure 5:**
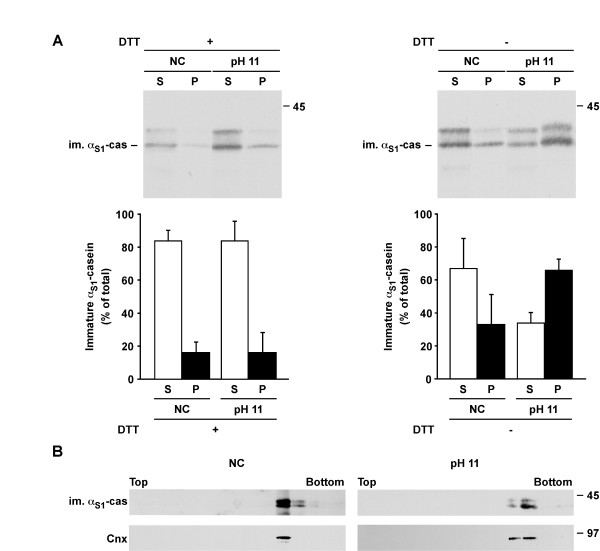
**The dimeric form of immature α_S1_-casein binds to ER membranes more efficiently**. A. Aliquots of the microsomes were diluted and incubated in non-conservative buffer (NC) or were subjected to carbonate extraction at pH 11 (pH 11), both with saponin, in the absence (-DTT) or the presence of 5 mM DTT (+DTT) and centrifuged. Supernatants (S) and pellets (P) were analyzed by SDS-PAGE followed by immunoblotting with anti-mouse milk proteins. The quantity of immature α_S1_-casein in the supernatant and the pellet was quantified by densitometry and expressed as percent of the total quantity of the casein (supernatant + pellet). The mean ± s.d. of three independent experiments is shown. B. Microsomes were treated as above, all in the presence of DTT, and extracted membranes were floated using linear sucrose gradient. Fractions were collected from the top (light sucrose) to bottom (heavy sucrose) and analyzed by SDS-PAGE followed by immunoblotting for the indicated markers. Representative gradients from two independent experiments are shown. Relative molecular masses (kDa) are indicated. Cnx: calnexin, im. α_S1_-cas: immature α_S1_-casein.

To investigate this possibility we further characterised the molecular form of α_S1_-casein that remained associated with the ER membranes. When microsomes were permeabilised in non-conservative conditions or after carbonate extraction at pH 11 with saponin, all in the presence of DTT, a similar if not identical proportion of α_S1_-casein (≈ 16%) was recovered with the membranous fractions (Figure 5A, left panel). In the absence of DTT the amount of α_S1_-casein in the pellet was higher, its quantity being superior in samples treated with carbonate at pH 11 (Figure 5A, right panel). It should be noted that the vast majority of β-casein (≥ = 95%) was found in the soluble fraction in both sets of experiments (data not shown). To further prove that the α_S1_-casein found in the membrane fraction was truly associated with membrane and not co-pelleting casein aggregates, extracted membranes were subjected to flotation using linear sucrose gradient (Figure 5B). After centrifugation at equilibrium, a small proportion of α_S1_-casein was recovered in the load and the large majority floated in the bottom fractions of the linear sucrose gradient. As attested by co-fractionation with calnexin, these fractions contained the extracted membranes. Both α_S1_-casein and calnexin had a similar behaviour in the absence of DTT (data not shown). These data demonstrated that a fraction of α_S1_-casein exists as a membrane-associated form within microsomal vesicles and indicated a role for disulphide bonds in the interaction of the protein with membranes.

**Figure 6:**
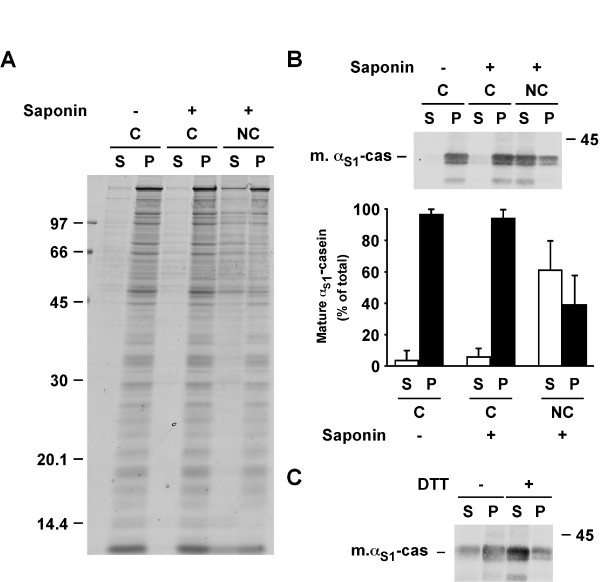
**Mature α_S1_-casein binds to post-ER membranes of the secretory pathway**. A, B. Aliquots of PNS were diluted and incubated in conservative (C) buffer in the absence of saponin (-) or in conservative or non-conservative (NC) buffers in the presence of saponin (+) and centrifuged. Supernatants (S) and pellets (P) were analyzed by SDS-PAGE. A. Coomassie Blue staining. B. Immunoblotting with an antibody against mouse milk proteins. The quantity of mature α_S1_-casein in the supernatant and the pellet was quantified by densitometry and expressed as percent of the total quantity of the casein (supernatant + pellet). The mean ± s.d. of three independent experiments is shown. C. Aliquots of PNS were subjected to carbonate extraction at pH 11 with saponin in the absence (-) or in the presence (+) of DTT and centrifuged. Supernatants and pellets were analyzed by SDS-PAGE followed by immunoblotting as above. Data are representative of at least three independent experiments. Relative molecular masses (kDa) are indicated. m. α_S1_-cas: mature α_S1_-casein.

To study the membranous form of α_S1_-casein in more distal compartments of the secretory pathway, aliquots of the membranes prepared from PNS, and including the whole secretory pathway, were treated as described above. After incubation in conservative conditions, few proteins were found in the supernatant (Figure 6A) and some additional bands were detected following permeabilisation by saponin. In non-conservative conditions, the proportion of these proteins in the supernatant was increased and other proteins appeared. Quantification of mature α_S1_-casein revealed that the majority of the protein (94.33%) remained associated with the membrane pellet after permeabilisation in conservative conditions. As expected, much more mature α_S1_-casein was released into the supernatant (61%) in non-conservative conditions. However, compared to immature α_S1_-casein in heavy microsomes (see Figure 4D), the proportion of the mature protein remaining associated with the PNS-derived membranes in non-conservative conditions doubled. Furthermore, PNS membranes were treated with carbonate buffer at pH 11 with saponin, in the absence or the presence of DTT. Figure 6C shows that a larger fraction of mature α_S1_-casein was recovered in the pellet in the absence of DTT, in agreement with our results in microsomes. These data demonstrated the presence of membrane-associated mature α_S1_-casein in distal compartments of the secretory pathway and confirmed the importance of disulphide bonds in the behaviour of this protein toward membranes.

**Figure 7:**
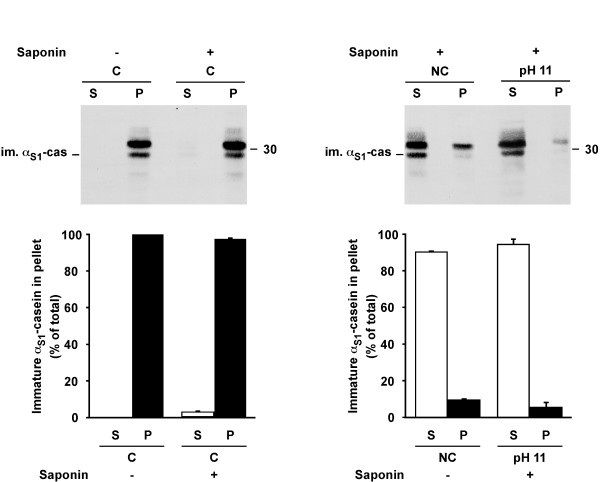
**α_S1_-casein is present in a membrane-associated form in rough ER microsomes from goat mammary gland tissue**. Aliquots of the microsomes prepared from goat mammary gland tissue were diluted and incubated in conservative (C) buffer in the absence of saponin (-), in conservative or non-conservative (NC) buffers in the presence of saponin (+), or were treated with carbonate buffer at pH 11 in the presence of saponin (pH 11). After centrifugation, supernatants (S) and pellets (P) were analyzed by SDS-PAGE followed by immunoblotting with an antibody against goat α_S1_-casein. Immature α_S1_-casein was quantified by densitometry and the proportion of the immature form in the supernatants and the pellets were expressed as percent of the total quantity of the casein (supernatant + pellet). The means of the results obtained from two animals are shown; bars indicate the variation of the single values from the mean. Relative molecular masses (kDa) are indicated. im. α_S1_-cas: immature α_S1_-casein.

Finally, we wished to generalize our observations to ruminants. Goat was chosen because the various forms of goat caseins was previously characterised by our group [9]. The permeabilisation experiments and extraction procedures were applied to goat rough ER microsomes. In comparison to the results in rat, we observed more mature α_S1_-casein in the microsomal fraction, most probably due to higher level of contamination by milk caseins contained in the goat tissue (Figure 7). However, as previously observed in rat, the vast majority of immature α_S1_-casein was recovered in the pellet after membrane permeabilisation in conservative conditions (Figure 7 left panel). Permeabilisation in non-conservative conditions or extraction with carbonate at pH 11 revealed that a small proportion of immature α_S1_-casein (5-10%) was also present in a tightly membrane-associated form in goat.

## Discussion

To elucidate the role of individual caseins in the aggregation process and in the secretion of the micelle, we wished to characterise the initial casein aggregate formed in the secretory pathway. In the present study, we focused on the putative aggregation events that occur in the ER for several reasons. First, it is well established that secretory proteins concentrate by up to 100-200-fold between the ER and the cis-Golgi [29-31], the ER export machinery being selective in sorting cargo [32]. Second, caseins that contain cysteine residues dimerise via disulphide bonds [14] and the ER is the presumptive site of disulphide bond formation. Third, we hypothesized that caseins interact in the ER because, in the absence of α_S1_-casein, β- and κ-casein accumulate in the ER due to a drastic reduction of caseins exiting from that compartment [9].

Morphological and biochemical controls demonstrated that the isolated rough ER fraction obtained from rat or goat mammary gland tissues was almost pure and hardly contaminated with casein micelles. In addition, focusing our analysis on the immature ER forms of α_S1_- and β-caseins (in rat only for the latter) allowed us to specifically monitor the caseins within the lumen of the ER. We would have liked to obtain similar information for κ-casein, but this was hampered by the lack of relevant immunological tools for rat κ-casein. Moreover, the immature form of this protein has not yet been identified. However, preliminary experiments in goat suggest that immature κ-casein behaves similarly to immature α_S1_-casein (data not shown).

It has been reported that amphiphilic bovine β-casein has the ability to self-associate into micelles in vitro [33,34]. This aggregation into oligomers is spontaneous, reversible and dependent on various parameters including temperature, ionic strength and protein concentration [2,35-39]. Moreover, mixed associations of β-casein with α_S1_-casein in conditions close to that of the ER have been obtained in vitro [40]. The observation that only few chains of immature β-casein remained in the microsomal membrane pellet after saponin permeabilization in conservative conditions suggested that this casein was either weakly associated with primary casein aggregates or was not prone to aggregation in the ER. This, in turn, may suggest that β-casein has no specific role in the initial step of casein micelle formation. In agreement with this, Kumar et al. [10] showed that formation of casein micelles still occurred after knock-out of the β-casein gene in mouse, micelles lacking β-casein only having a smaller diameter than control ones. Similarly, casein micelles were observed in the secretory pathway of MECs from goats that do not express β-casein (β-Cn0/0 goat, [9]). In both cases, casein transport in the secretory pathway and secretion were apparently not affected. Moreover, it is also well-known that β-casein easily detaches from casein micelles, e.g. upon cooling of milk [41], whereas interactions between the other caseins are much stronger. In line with this, it should be noted that our permeabilisation experiments were performed at 4°C to avoid proteolysis during incubation. Notably, it has been demonstrated that the phosphorylation of β-casein occurs in a later Golgi compartment than that of α_S1_-casein [16-18]. Moreover, since bovine non-phosphorylated β-casein was shown to have a different micellisation process than the phosphorylated form [42], we can hypothesise that the mature and immature forms of rat β-casein have different association properties. The observation that, after permeabilisation in conservative conditions, the proportion of the mature form of the protein in the membrane pellet derived from a PNS was higher than that of the immature form in the microsomal pellet (data not shown) was consistent with this hypothesis. We concluded that rat β-casein might interact efficiently with the other components of the micelle in a more distal compartment of the secretory pathway, may be after its phosphorylation when it meets high calcium concentration in late Golgi cisternae and/or secretory vesicles.

In contrast to β-casein, the majority of immature α_S1_-casein was found in the membrane pellet after permeabilisation in conservative conditions, in both rat and goat microsomes. Notably, a substantial proportion of this protein remained associated with microsomal membranes after permeabilisation in non-conservative conditions or extraction with carbonate at pH 11. These results demonstrated the presence of soluble (at most 25% in rat), aggregated and membrane-associated forms of α_S1_-casein in the rough ER microsomes. Consistent with this, in vitro experiments led to the conclusion that α_S1_-casein can self-associate [43,44], the two hydrophobic regions of bovine α_S1_-casein interacting to form a polymeric chain (for review see [6]). In addition, bovine α_S1_-casein was also found to prevent κ-casein aggregation and accumulation of κ-casein fibrils [45-47], as well as to reduce β-casein aggregates in vitro [40], features that might be relevant for casein micelle formation. Also, Bouguyon et al. [14] demonstrated that mature α_S1_-casein from rat milk can form an intermolecular disulphide bond. Finally, in vivo experiments in goat revealed that α_S1_-casein might interact with the other caseins in the ER to facilitate the export of these proteins to the Golgi apparatus [9]. It is therefore tempting to speculate that α_S1_-casein acts as an escort protein (or "transport chaperone", [48]) for efficient packaging of the other caseins into ER-derived transport carriers and transport to the Golgi. In the absence of α_S1_-casein, however, casein micelles still form. Altogether, these data are consistent with the fact that a large proportion of immature α_S1_-casein is aggregated in the rat rough ER through covalent and non-covalent interactions with itself or with other caseins. We conclude that, in contrast to β-casein, α_S1_-casein most likely participates in a primary casein aggregate which forms in the ER prior to its transport to the Golgi apparatus. This differential behaviour of β-casein and α_S1_-casein might play a key role in the spatio-temporal dimension of casein micelle formation within the secretory pathway, delaying formation of big aggregates to the late steps of transport within secretory vesicles.

We found that a substantial proportion of α_S1_-casein was strongly interacting with the ER membranes. We also detected a membranous form of α_S1_-casein in pH 11 extracted membranes prepared from rabbit MECs (data not shown). Moreover, we found α_S1_-casein in a proteomic analysis of rough ER microsomal membranes prepared from goat (H. Lahouassa, manuscript in preparation). Of note, the proportion of membrane-associated α_S1_-casein was higher in rat (≈ 15%) than in the other mammals studied so far (≈ 5-10%). However, the latter proportion is similar to those found for other secretory proteins known to interact with membranes (see below). In rat, the structural domain of α_S1_-casein involved in this interaction might therefore have a higher affinity for its binding site. Consistent with our observation, an in vitro study with liposomes proved interactions between bovine milk caseins (κ-casein oligomers and early globular casein aggregates) and membrane phospholipids [49].

Membranous forms of secretory proteins have been observed in several instances, including hormones (for review see[50]), prohormone convertases and processing enzymes [51,52] and members of the granin (chromogranins, secretogranins) family of regulated secretory proteins [53,54] that are ubiquitously found in secretory granules of neuroendocrine and neuronal cells (for review see [55]). Concerning the latter proteins, it was proposed that the membranous form of these proteins is a "nucleus" for granin aggregation in the trans-Golgi network (TGN), a process required for targeting of these proteins to secretory granules. The membrane-associated forms of the granins can therefore be considered sorting receptors. In the context of casein micelle formation, we also hypothesize that the membranous form of α_S1_-casein acts as a "nucleus" for casein association/aggregation in the ER for further targeting of the other caseins to the site of COP II vesicle formation. Casein micelles are often found attached to the membranes of secretory vesicles through electron dense proteinaceous material most likely corresponding to condensates of casein molecules (see figures in [19,20,40]). Consistent with this hypothesis, spherical particles are also seen in close apposition to the saccular membranes in the Golgi apparatus.

We observed that dimerisation played a key role in the interaction properties of α_S1_-casein with rough ER microsomal membranes. In line with this, we showed that rat caseins are actually dimeric in rough ER microsomes, as expected since the ER is the presumptive site for disulphide bond formation. From this, we can conclude that dimerisation is the first step in casein micelle formation. For, chromogranin B, a disulphide-bonded loop in its N-terminal sequence was shown to play a key role at the level of the TGN in its sorting to secretory granules [56]. Subsequently, it has been demonstrated that the disulphide-bonded loop mediates homodimerisation of chromogranin A [57], and most likely B, as well as their association with membranes [58]. Moreover, their data strongly suggest that when the protein aggregates in the TGN, this cargo with multiple loops on its surface has a high membrane binding capacity, a feature important for efficiency of sorting to secretory granules. Our experiments using total membranes from PNS revealed the existence of a membranous form of mature α_S1_-casein and confirmed the role of disulphide bonds in the association of the protein with these membranes. This implies that α_S1_-casein is able to also interact with membranes of downstream compartments of the secretory pathway. Our observation of a higher proportion of membrane-associated α_S1_-casein in PNS also suggests that phosphorylation of the protein and/or lipid composition of the membranes might be involved in membrane interaction.

## Conclusions

In the present study we report for the first time the existence of a membrane-associated form of a casein, specifically α_S1_-casein. We believe that this form of the protein may play a key role in both casein micelle formation and casein transport in the secretory pathway. First, membrane-associated α_S1_-casein might serve as a nucleating site for the aggregation of the caseins and growth of the micelle. Second, this form might also have a function in concentration of the cargo and sorting of the caseins for packaging into COPII vesicles and export from the ER. The possibility that membrane-associated α_S1_-casein is involved in subsequent steps of casein aggregates and/or casein micelle sorting and transport in the secretory pathway remains to be studied.

## Methods

### Animals and antibodies

Wistar rats, raised in our institute (Nutrition et Régulation Lipidique des Fonctions Cérébrales, INRA, Jouy-en-Josas, France), were used at mid-lactation. Euthanasia was by decapitation. French-Alpine goats homozygous at the *CSN1S1 *casein locus (allele A) were obtained from INRA, UE332 Domaine experimental de Bourges (La Sapinière, France). Allele A is associated with high α_S1_-casein content in milk. Genotypes were determined using a PCR-based allele-specific typing procedure [59]. Goats were fasted for 24 hours, injected with oxytocin, milked and euthanatized. Animal welfare and conditions for animal handling were in accordance with French guidelines (May 2001). Antibodies against mouse whole milk proteins (RAM/MSP) were from Nordic Immunological laboratories (Tilburg, The Netherlands) and used at a dilution of 1:2500 for immunoblotting. The monoclonal antibody against protein disulphide isomerase (PDI) and the rabbit polyclonal antibodies against immunoglobulin heavy chain binding protein (GRP78/BiP) and against vesicle-associated membrane protein 4 (VAMP4) were from Abcam^® ^and used at a dilution of 1:5000, 1:10,000 and 1:1000, respectively. Rabbit polyclonal antibodies against calreticulin and calnexin were purchased from StressGen Biotechnologies Corp. (Victoria, BC, Canada) and used at a 1:1000 dilution. Rabbit antiserum against goat α_S1_-casein was obtained from M.-F. Mahé (Jouy-en-Josas, France) and used at a dilution of 1:5000. HRP-conjugated secondary antibodies were from goat (anti-rabbit, Jackson Immunoresearch Lab., Inc., Avondale, PA, USA) or from sheep (anti-mouse, Sigma-Aldrich) and used at a dilution of 1:5000 or 1:2000, respectively. Unless otherwise indicated, chemicals were from Sigma-Aldrich or Research Organics.

### Preparation of the rough ER microsomal fraction

Total rough ER microsomes were prepared from rat or goat mammary gland homogenate by differential centrifugation followed by sucrose density gradient as described by Paiement et al. [23], with some minor modifications. For rat, mammary glands were removed and transferred to ice-cold 0.25 M sucrose. All subsequent steps were performed at 4°C. Samples were dissected free from connective tissue and muscles, and finely chopped into ≈1-2 mm^3 ^pieces using scissors. Mammary gland fragments were washed 3 times for 10 minutes in 0.25 M sucrose to remove milk constituents and further minced using a homemade multi-mounted razor blade device. Tissue was homogenized using a 20 ml Teflon-glass homogenizer (BB, Thomas scientific) for 3 strokes. The homogenate was filtered through a piece of 150 μm polypropylene mesh (ZBF, Rüschlikon, Switzerland) and centrifuged at 8700 g in a Beckman JS 13.1 rotor for 13 minutes. The resulting supernatant, also referred to as post-nuclear supernatant (PNS), was centrifuged at 43,000 g in a TI 50.2 rotor for 6 minutes and 40 seconds (once maximal speed is reached, stop with the break). The supernatant (S2) and the flocculent upper layer of the pellet (P2) were collected and centrifuged at 110,000 g in a TI 50.2 rotor for 1 hour. The supernatant was collected (S3) and the pellet (P3) was resuspended in 2.0 M sucrose, homogenized using a Dounce, and diluted to obtain a final sucrose concentration of 1.38 M. The homogenate was loaded into a centrifuge tube, layered with a step-gradient (1.0, 0.86 and 0.25 M sucrose) and centrifuged in a Beckman SW55 TI rotor at 300,000 g for 1 hour. In some experiments the various sucrose fractions were pooled (S4) and an aliquot was analyzed by SDS-PAGE. The pellet, which contains the rough ER microsomes, was resuspended in 2 mM imidazole pH 7.4, 0.25 M sucrose, homogenized using a Dounce and centrifuged at 140,000 g in a Beckman 50 TI rotor for 1 hour. The final rough ER microsomal pellet (M) was resuspended in 2 ml of 2 mM imidazole pH 7.4, 0.25 M sucrose and aliquots were stored at -80°C. For preparation of goat rough ER microsomes, the first centrifugation was at 6000 g for 15 minutes. Protein concentration in fractions was determined.

### Milk fractionation

Wistar rats at mid-lactation were injected with oxytocin (0.6 ml oxytocin at 5 UI/ml) a few minutes before anaesthesia (200 μl pentobarbital at 6 g/100 ml) and milk was collected manually. Samples were aliquoted and stored at -80°C. For analysis, aliquots were thawed and kept at room temperature for 1 hour before taking samples from the aqueous phase, below the cream layer. All subsequent steps were performed at room temperature. Samples were diluted 10 times in conservative (10 mM Hepes-KOH pH 6.8) or non-conservative (25 mM Hepes-KOH pH 7.4; 150 mM NaCl, 20 mM EDTA, 0.3% Tween 20 (v/v), 5 mM DTT) buffer and incubated for 15 minutes under rotating wheel gyratory shaking. After centrifugation at 16,000 g for 15 minutes, supernatants and pellets were analyzed by SDS-PAGE (see below) followed by Coomassie blue staining or immunoblotting. For negative staining analysis in electron microscopy, milk aliquots were either directly processed or incubated as above.

### Membrane permeabilisation

All steps were performed at 4°C. Aliquots (50 μg protein for rat or 100 μg for goat) of the microsomal fraction or of the membrane-bound organelles prepared from PNS were diluted ≈ 10-15 fold (final volumes: 150 μl for rat or 300 μl for goat) in either conservative (10 mM Hepes pH 7) buffer in the absence or the presence of 0.1% saponin (w/v), non-conservative or carbonate (100 mM Na_2_CO_3 _pH 11, 1 M KCl, 2 mM EDTA, 5 mM DTT) buffer containing 0.1% saponin [60]. In some experiments, DTT was omitted. All solutions were supplemented with a protease inhibitor cocktail (Sigma-Aldrich). Samples were subjected to a 30-minute incubation followed by centrifugation at 110,000 g for 1 hour. Membrane pellets obtained after incubation in conservative or non-conservative conditions were washed for 15 minutes in 10 mM Hepes pH 7 and centrifuged as above. Proteins in supernatants were subjected to TCA precipitation (10% final concentration) for 1 hour. In the case of carbonate treatment, membrane pellets were subjected to a second round of incubation in carbonate buffer for 15 minutes, centrifuged and the resulting membranes were washed for 15 minutes in 10 mM MES pH 6.5. Carbonate supernatants were neutralised using 1 N HCl and subjected to TCA precipitation for 1 hour. Equivalent amounts of supernatant or pellet aliquots were analyzed by SDS-PAGE followed by Coomassie blue staining or immunoblotting.

### Sucrose density gradient

Rat microsomal membranes (100 μg protein) were permeabilised by saponin either in non-conservative conditions or in carbonate buffer, both in the presence of DTT, and centrifuged as described above. The resulting membrane pellets were subjected to flotation using sucrose density gradient according to Schuck et al. [61] with minor modifications. All steps were performed at 4°C. Extracted membranes were resuspended in 500 μl of 25 mM Hepes-KOH pH 7.4, 150 mM NaCl, 2 mM EDTA, supplemented with a protease inhibitor cocktail (Sigma-Aldrich), and incubated for 30 minutes. Samples were adjusted to 40% sucrose with 1 ml of 60% sucrose, transferred into centrifuge tubes, overlaid with 11 ml of a 5-30% linear sucrose gradient in the above buffer and centrifuged at 270,000 g in a Beckman SW41 rotor for 17 hours. All sucrose solutions were in the above buffer. Fractions (1 ml) were collected from the top, proteins subjected to TCA precipitation for 1 hour, and analyzed by SDS-PAGE followed by immunoblotting.

### Electrophoresis and immunoblotting

Samples were analyzed by SDS-PAGE using 12% gels according to Laemmli [62], except that the sample buffer contained 3 mM EDTA. In some experiments, the reducing agent (3.3% β-mercaptoethanol) was omitted. Gels were stained and destained or subjected to Western blot using Hybond-C Extra (GE Healthcare Life Sciences). Membranes were blocked in polyvinyl alcohol (1 mg/ml) in phosphate buffer saline (PBS) for 1 minute. Incubations with antibodies were in PBS plus 0.3% Tween 20 (v/v) and 10% skim milk powder (w/v). Antigens were revealed by enhanced chemiluminescence (ECL) western blotting detection reagents (GE Healthcare, Buc, France) and X-ray film (XBM Retina, Onde & Rayons, France). When necessary, membranes were stripped using the Restore™ western blot stripping buffer (Pierce, Rockford, IL, USA).

### Electron microscopy

For electron microscopy, samples were fixed with 2% glutaraldehyde in 0.1 M Na cacodylate buffer pH 7.2 for 1 hour at room temperature or 15 hours at 4°C. Samples were postfixed with 1% osmium tetroxide containing 1.5% potassium cyanoferrate, gradually dehydrated in ethanol (30% to 100%) and embedded in Epon which was polymerized at 60°C. Thin sections (70 nm) were collected onto 200 mesh cooper palladium grids, and counterstained with lead citrate.

For negative staining analysis, an aliquot of milk or of milk diluted in conservative or non-conservative buffer were applied to 300 mesh carbon-coated copper grids. After 1 minute, excess liquid was removed and grids were contrasted using 1% uranyl acetate.

Samples were observed on a Zeiss EM 902 transmission electron microscope at 80 kV. Images were acquired using a MegaView III CCD camera and analyzed with ITEM software (Eloïse, SARL, Roissy CDG, France).

### Quantification

The intensity of the ECL signal for relevant protein bands was quantified from X-ray film scans (600 dpi) using the Image J software (Wayne Rasband, NIH, USA, http://rsb.info.nih.gov/ij/). The background noise was estimated in the proximal area of the film and subtracted from the integrated density of the protein band.

To estimate the purity of rat rough ER microsomes in the corresponding subcellular fraction, the relative proportion of immature caseins was quantified. The amounts of the immature or mature form of rat α_S1_- and β-casein were determined as described above and for each casein, the ratio of the immature form to total casein (immature plus mature) was calculated and expressed in percent.

To monitor the behaviour of the caseins after permeabilisation in conservative or non-conservative conditions, as well as upon carbonate treatment, the amounts of the immature form of rat α_S1_- and β-casein or the immature form of goat α_S1_-casein in the supernatant or in the pellet were calculated. For each casein, the proportion in the pellet was estimated by the ratio of the quantity of the immature form in the pellet to the total quantity of the casein (pellet plus supernatant) and expressed in percent.

Protein concentrations were determined using the Peterson procedure [63] with bovine serum albumin as the standard.

To estimate the mean size of microsomes, the membranes of the structures contained in 1500 nm^2 ^were manually drawn using the ITEM software which calculates the diameters.

## Abbreviations

Cnx: calnexin; Crt: calreticulin; ECL: enhanced chemiluminescence; ER: endoplasmic reticulum; GRP78/BiP: immunoglobulin heavy chain binding protein; MECs: mammary epithelial cells; PBS: phosphate buffer saline; PDI: protein disulphide isomerase; PNS: post-nuclear supernatant; TGN: trans-Golgi network; VAMP4: vesicle-associated membrane protein 4.

## Authors' contributions

ALP participated in the design of the study, conducted all the experiments presented and helped to draft the manuscript. JL and EC conceived the study, supervised the work and provided continuous guidance. EC participated in the design of the study and formulated the final draft of the paper. All authors have read and approved the final manuscript.
